# Prednisolone Versus Colchicine for Acute Gout in Primary Care: statistical analysis plan for the pragmatic, multicenter, randomized, and double-blinded COPAGO non-inferiority trial

**DOI:** 10.1186/s13063-024-08066-0

**Published:** 2024-04-03

**Authors:** Adrian Richter, Julia Truthmann, Eva Hummers, Julia Freyer Martins Pereira, Ildikó Gágyor, Franziska Schuster, Amelie Witte, Susanne Böhm, Alexandra Greser, Petra Kamin, Sylvia Stracke, Marcus Dörr, Robin Bülow, Stefan Engeli, Jean François Chenot, Till Ittermann

**Affiliations:** 1 Department of Prevention Research and Social Medicine, Institute for Community Medicine, University Medical Center Greifswald, Walther-Rathenau-Str. 48, 17475 Greifswald, Germany; 2 Department of General Practice, Institute for Community Medicine, University Medical Center Greifswald, Greifswald, Germany; 3 Department of General Practice, University Medical Center Göttingen, Göttingen, Germany; 4 Department of General Practice, University Hospital Würzburg, Würzburg, Germany; 5 Coordinating Center for Clinical Studies, University Medical Center Greifswald, Greifswald, Germany; 6 Department of Internal Medicine A, Nephrology, University Medical Center Greifswald, Greifswald, Germany; 7 Department of Internal Medicine B, Cardiology, University Medical Center Greifswald, Greifswald, Germany; 8 Institute for Radiology and Neuroradiology, University Medical Center Greifswald, Greifswald, Germany; 9 Institute of Pharmacology, University Medical Center Greifswald, Greifswald, Germany; 10 Department SHIP-KEF, Institute for Community Medicine, University Medical Center Greifswald, Greifswald, Germany

## Abstract

**Background:**

To date, colchicine and prednisolone are two effective therapies for the treatment of acute gout but have never been compared directly in a randomized clinical trial. In addition, in previous trials of treating acute gout patients with concomitant comorbidities were often excluded due to contraindications to naproxen.

**Study design:**

This pragmatic, prospective, double-blind, double-dummy, parallel-group, randomized, non-inferiority trial compares prednisolone with colchicine in terms of non-inferiority in patients with acute gout. Patients presenting to their general practitioner with acute gout can be included if the gout attack has occurred within the last 2 days. A total of 60 practices in the vicinity of three university medical centers (Greifswald, Göttingen, and Würzburg) participate in the study. The intervention group receives 30 mg prednisolone for 5 days, while the group of standard care receives low-dose colchicine (day 1: 1.5 mg; days 2–5: 1 mg). The first dose of treatment is provided at day 0 when patients present to the general practitioner due to an acute gout attack. From day 0 to day 6, patients will be asked to complete a study diary on daily basis regarding pain quantification. For safety reasons, potential side effects and the course of systolic blood pressure are also assessed.

**Statistical analysis plan:**

*N* = 314 patients have to be recruited to compensate for 10% of dropout and to allow for showing non-inferiority of prednisolone compared to colchicine with a power of 90%. We use permuted block randomization with block sizes of 2, 4, and 6 to avoid imbalanced treatment arms in this multi-center study; patients are randomized in a 1:1 ratio. The absolute level of pain on day 3 (in the last 24 h) is the primary outcome and measured on a numerical rating scale (NRS: 0–10). Using a multiple linear regression model adjusted for age, sex, and pain at baseline, prednisolone is considered non-inferior if the effect estimate including the confidence intervals is lower than a margin of 1 unit on the NRS. Average response to treatment, joint swelling and tenderness, physical function of the joint, and patients’ global assessment of treatment success are secondary outcomes.

**Discussion:**

The trial will provide evidence from a direct comparison of colchicine and prednisolone regarding their efficacy of pain reduction in acute gout patients of primary care and to indicate possible safety signals.

**Trial registration:**

ClinicalTrials.gov Identifier: NCT05698680 first posted on January 26, 2023 (retrospectively registered).

## Introduction

### Background and rationale (7)

The updated EULAR recommendations (2016) include colchicine as a first-line treatment option for acute attacks of gout disease [1]. In contrast, the national guideline of general practitioners (GPs) and family physicians (DEGAM) in Germany considers the use of prednisolone as a line-line option for the treatment of acute gout, due to concerns about the effectiveness of colchicine [2].

In addition to controversial recommendations, there is only low-quality evidence for use of colchicine, and the drug has been associated with serious adverse events [3], including death, mostly due to accidental overdosing [4]. Direct comparisons of colchicine and prednisolone for the treatment of acute flares in gout are lacking which contributes to no established consensus. The need for a randomized controlled clinical trial comparing colchicine and corticosteroids has been repeatedly mentioned [1, 5, 6].

### Objective (8)

The objective is to compare the efficacy of oral prednisolone versus colchicine in patients with an acute gout flare treated in primary care setting.

### Research hypotheses

In this non-inferiority trial, we examine whether prednisolone (test treatment (TT)) is acceptably worse than treatment with colchicine (standard treatment (ST)). Therefore, we will compare as our primary outcome the absolute levels of the most severe pain (last 24 h) measured with an 11-point numeric rating scale at day 3 after baseline. The hypotheses are as follows:

*H*_0_: ST is superior to TT in terms of mean pain at day 3 of follow-up *μ*_*ST*_ − *μ*_*TT*_ ≤  − *δ*_*NI*_

*H*_1_: TT is non-inferior to ST in terms of mean pain at day 3 of follow-up *μ*_*ST*_ − *μ*_*TT*_ >  − *δ*_*NI*_

*δ*_*NI*_ is the non-inferiority margin, and *μ*_*TT*_ (*μ*_*ST*_) is the mean pain obtained under prednisolone (colchicine). The null hypothesis implies that treatment with colchicine is superior to the treatment with prednisolone. Accordingly, we formulate the alternative hypotheses that treatment with prednisolone is non-inferior to the treatment with colchicine.

## Study methods

### Trial design (9)

This trial is a multi-center, pragmatic, double-blind, parallel-group randomized non-inferiority trial comparing two approved treatments for acute gout.

### Randomization (10)

Randomization is applied in a 1:1 ratio of patients to receive either prednisolone or colchicine. We decided for permuted block randomization (PBR) and against simple or complete randomization (CR), as recommended for larger trials (*N* > 200) [7]. The decision was made due to findings of considerable imbalance of treatment arms in multi-center, pragmatic trials with drop-out of recruiting centers [8], due to analytical results [9, 10], and due to results from a simulation study (please see below).

Since the trial has two arms of active and efficacious treatments, uses double-dummy blinding [11], identical blister for both drugs, similar route of administration of both drugs, and small and random block lengths, we consider the possibility of drug prediction by physicians or trained staff as minimal [12].

#### Implications of trial design

Overall, three University Medical Center (Greifswald, Göttingen, and Würzburg) organize > 20 recruiting centers each (GP practices) in the respective region. For each of the recruiting centers, an allocation sequence will be randomly generated to enable recruitment of up to 30 patients. The randomization list was generated with random blocks of length 2–6 using the R package *blockrand* [13].

We expect a dropout of recruiting centers in terms of being unable to recruit patients according to inclusion criteria or due to other reasons [8]. Furthermore, the number of recruited patients will be heterogeneous between recruiting centers.

#### Simulations

**Fig. 1:**
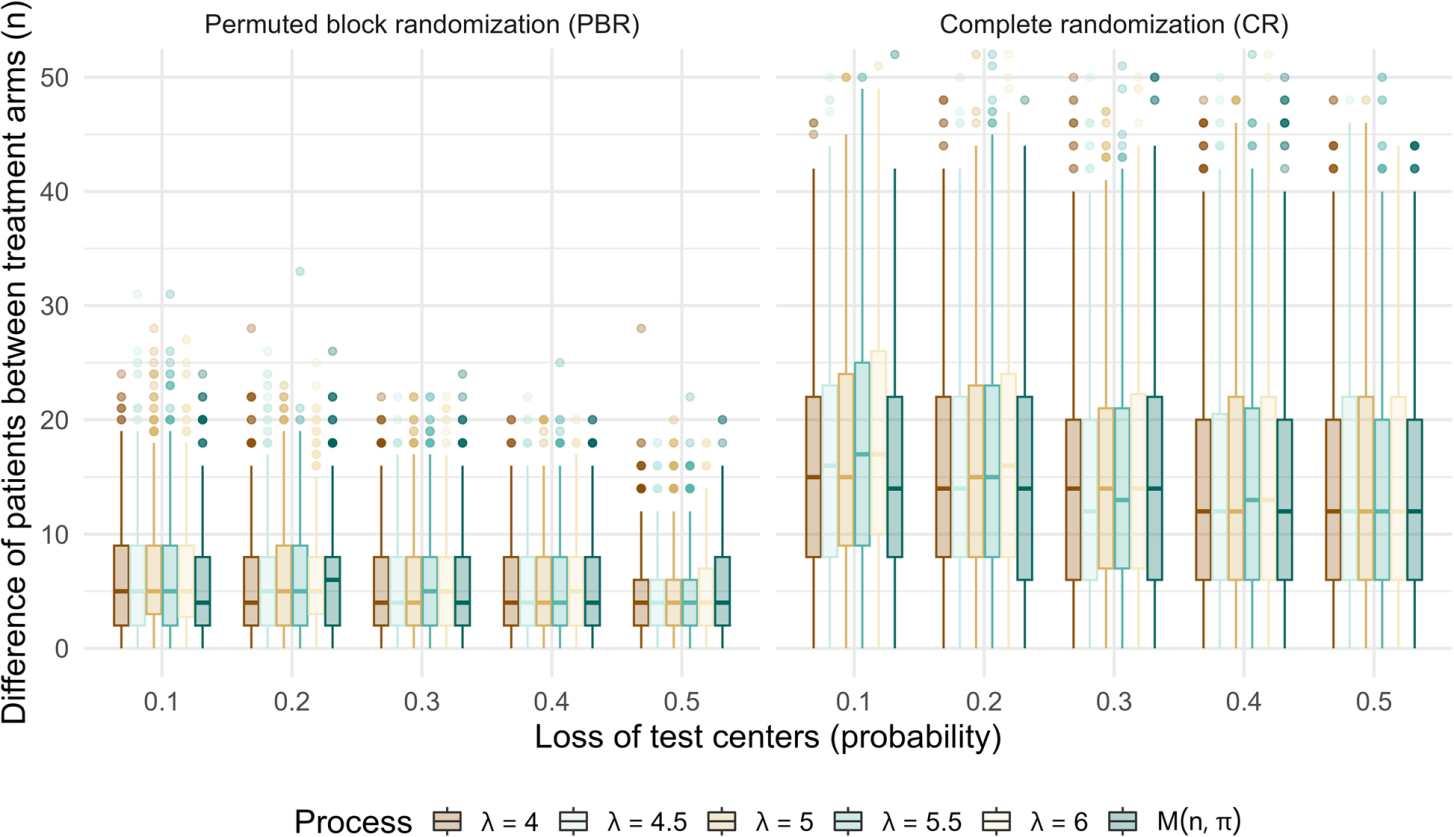
Simulation results of expected imbalance between treatment arms due to dropout and incomplete recruitment

Two simulation approaches were used to examine possible imbalance between treatment arms: ] (a) using an unrestricted zero-inflated Poisson distribution and (b) a restricted zero-inflated Poisson distribution, in which the overall sum of recruited patients is restricted to the required samples size. In both, zero-inflation is introduced by a binomial process to mimic varying probabilities of dropout of recruiting centers (10–50%). In the first approach (a), each recruiting center, that is not considered dropout, recruits’ patients according to a Poisson distribution *Poi*(*λ*_*i*_) and with *λ*_*i*_ varying between 4 and 6. The parameter vector is based on clinical experience from involved physicians who expected on average a recruitment of 4 to 6 patients within the recruitment period. The first approach is unrestricted and does neither guarantee to achieve the required sample size nor to avoid over-recruitment. In the second approach (b), the Poisson-part of the mixture distribution equals a multinomial distribution [14] since the sum over all recruited patients is *n* =  ∑ *λ*_*i*_. We applied 1000 random samples for each combination of the assumed dropout rates and the average expected number of recruited patients in approach 1 and, in approach 2, for each possible dropout rate. Predefined randomization lists were created for PBR using *blockrand* [13] and for CR using *randomizeR* (Fig. 1) [15].

The probability of an imbalance of greater than 20 patients was in most settings of PBR < 1% and on average > 25% using CR. Apparently, imbalance is almost independent from the mean of the Poisson distribution whereas a marginal decrease of the median imbalance is found for CR with increasing dropout of recruiting centers. Overall, imbalance is higher with CR and the results are in line with other studies [10].

#### Handling of randomization

As recommended by ICH E9 [16], the randomization list was generated by the sponsor. The study statistician created the R-script to generate the randomization list, but the setting of the seed was the responsibility of the sponsor. Principal investigators and the study statistician were blinded for the randomization list.

### Sample size (11)

According to American Pain Society, a 5–10-point improvement on a 100-point visual analogue scale (VAS) compares to a slight effect in pain relief [17]. This corresponds to a 0.5 to 1-point difference on a numerical-rating-scale (NRS) ranging from 0 to 10. The non-inferiority margin is therefore set to *δ*_*NI*_ = 1.

The treatment effect of prednisolone was studied in several trials [18–20] as well as for colchicine [21–23]. Nonetheless, no direct comparison of both drugs is available also not from observational studies. An indirect comparison of colchicine and prednisolone, both compared versus naproxen, is possible via the studies of Janssens et al. [18] (interval 7: 66–78 h) and Roddy et al. [23] (at day 3). Compared to naproxen, prednisolone (− 3%) showed slightly less efficacy than colchicine (− 2.2%; please see Fig. 2 in Roddy et al. [23] at day 3). However, naproxen in Janssens et al. [18] was given in higher dose (1 g/day) than in Roddy et al. (0.75 g/day) [23]. Therefore, we assume almost similar levels of pain measured on an NRS at day 3 of follow-up under both treatments; the difference in means of pain levels between treatments will likely not exceed 0.22 units on the NRS (0–10). Standard deviation at baseline measured on a VAS was 22.4 for prednisolone [18] and 2.2 for colchicine on a NRS [23]. Based on these two studies, we assume a common standard deviation of *σ* = 2.24 for sample size calculation in this study. Regarding dropout, Janssens et al. reported a dropout rate of less than 5% [18] and Roddy et al. [23] a dropout of 12.5% at day 7 of follow-up. Due to evaluation of the primary outcome pain at day 3 of follow-up in this trial, we assume a maximal dropout of 10%. Due to the short-term follow-up for the treatment of acute flares of gout, no assumptions regarding non-adherence to treatment allocation are assumed.

**Fig. 2:**
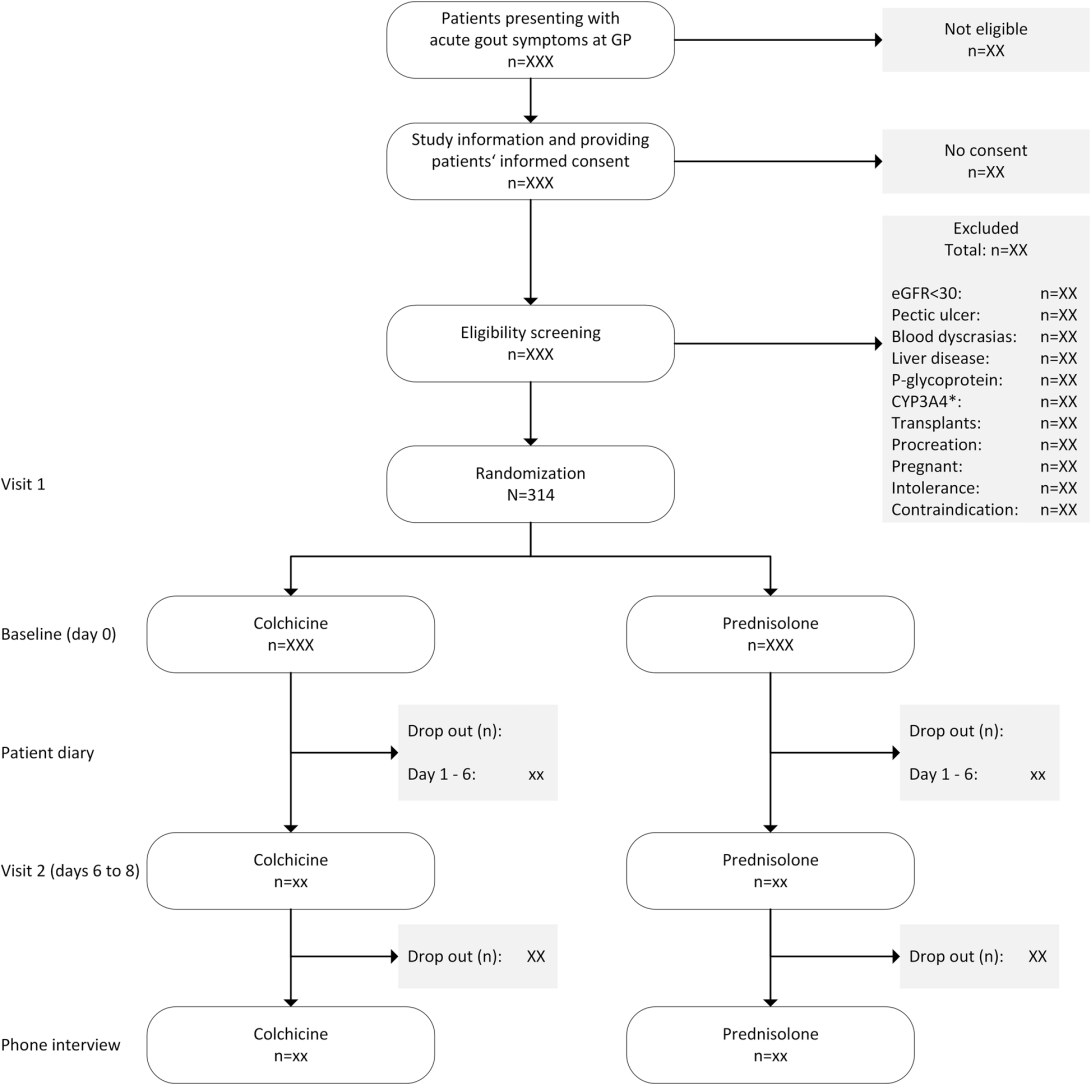
COPAGO trial flowchart

Sample size calculation is applied using a one-sided two-sample *t*-test, a significance level of 5%, and a power of 90%. The procedure PROC POWER of SAS version 9.4 (SAS Institute Inc., Cary, NC, USA) was used for this calculation. According to the assumptions made above, the sample size is *n* = 142 per treatment arm. Adding (ceiling of) 10% of patients assumed to drop out (*n* = 15) to each treatment arm results in a total sample size of 314 patients. Since a high number of protocol deviations is expected as in Roddy et al. [23] (≥ 30% of the study population), the trial is not powered to allow for overall conclusion on non-inferiority based on ITT-analysis and per-protocol-analysis. The required sample size does also not consider anticipated subgroup analyses.

### Framework (12)

The primary outcome is tested for non-inferiority of prednisolone. This applies also for the secondary outcome of the mean pain outcome over days 1 to 6 of follow-up. Remaining secondary outcomes are examined on an exploratory basis.

### Statistical interim analyses (13a)

No interim analysis will be conducted.

### Adjustment of significance level (13b)

No adjustment of the confidence level will be conducted.

### Stopping guidance (13c)

No statistical method will be applied to guide a stopping decision of the trial.

For the individual patient: in line with usual clinical care, the patients will have the possibility to terminate the treatment at any time without any disadvantage. All patients who discontinue a trial intervention will receive ongoing treatment according to the decision of the treating GP, unless unwilling to do so. Patients could be excluded during the trial due to occurring exclusion criteria, compliance violations or safety reasons (e.g., severe side effects).

For participating sites: the sponsor will be authorized to terminate participating sites due to safety or economic reasons, such as frequent major findings during monitoring or audits, low patient recruitment, or upcoming lack of qualified personnel. The principal investigator will be obligated to inform the sponsor immediately, if there is any evidence that may result in the closure of a participating site. Procedures like escalations and the protection of collected data will be defined in the site agreement.

For the whole trial: premature termination or suspension of this clinical study may occur according to the instructions of the national competent authority, ethics committee, or the sponsor/principal investigator.

### Timing of final analysis (14)

The database will be cleaned and locked after the last patient has completed all visits according to the study schedule. All analyses will be conducted thereafter.

### Timing of outcome assessment (15)

Measures of the primary and secondary outcome (pain, joint tenderness and joint swelling, blood pressure) are assessed at baseline and at days 1 to 6 of follow-up. Adverse events are assessed at the second visit at the GP between days 6 and 8, at the optional DECT examination (days 7–13), and during the telephone interview (days 27–34). The imaging using DECT is conducted between day 7 and 13 of follow-up.

At baseline (study visit 1), patients will receive a self-administered, standardized questionnaire and will be examined and interviewed by their GP, and a blood sample will be drawn. In addition, GPs will conduct a medical record review. Between study visits 1 and 2, patients will be asked to complete a patient diary with a restricted number of outcomes (pain (NRS), joint status, drug-related side effects, blood pressure). At study visit 2 (days 6–8 after baseline), patients will be re-assessed by the GP and return their patient’s diary, packages, and remaining trial medication for pill count. This varying follow-up time has been chosen as the end of the follow-up in this pragmatic trial conducted by GPs may involve public holidays. Patients may opt to undergo a DECT imaging once during days 7–13 after baseline at the university medical centers Göttingen and Greifswald. A phone interview will take place between days 27 and 34 after baseline to interview patients on the course of gout disease.

## Statistical principles

### Confidence intervals and *P* values (16)

For the primary outcome the applied tests will be two-sided using a significance level of *α* = 0.05. All results will be shown including effect estimates and 95% confidence intervals. For the analysis of adverse events, rates including Clopper-Pearson exact confidence intervals [24] will be used since a low number of events is assumed.

### Multiplicity corrections (17)

No corrections for multiple testing will be applied.

### Confidence intervals to be reported (18)

95% confidence intervals will be reported for all effect estimates.

### Adherence and protocol deviations (19a-d)

Compliance with the trial intervention is defined as taking 100% of the trial medication until day 4 of follow-up. Therefore, the number of pills is counted at the second visit at the GP. The percentage of compliant patients will be analyzed for each trial arm (*N*, percent) and considered in a respective sensitivity analysis.

The following list issues deviations or non-adherence from the study protocol:

- Incomplete use of medication
- Subsequent realization of exclusion criteria according to paragraph 8.3 of the study protocol
- Acute gout flare lasted longer than 48 h* prior initial medication
- A different underlying disease has been noticed, e.g., septic arthritis
- Attrition prior day 6

* prior amendment October 10, 2023: 24 h

### Analysis populations (20)

The statistical evaluation of the primary outcome will be conducted according to the *intention-to-treat* principle (ITT). In non-inferiority trials, ITT analyses may be considered anti-conservative [25]. Nevertheless, a recent systematic review has shown the opposite [26]. We consider the possibility of this limitation though and will report per-protocol results. Sample size calculations are adapted to the ITT approach and consider only attrition from the trial.

We do not differ a safety from an efficacy population as the true exposure to one of the drugs is hardly safeguarded. Therefore, all patients randomized are included in the efficacy and safety analyses. Missing data will be imputed to ensure analyses of all patients. Please see the paragraph regarding the handling of missing data in this SAP. For sensitivity analysis of the primary outcome, a *per-protocol population* will be defined as those participants adhering to the study protocol medication plan until day 3 of follow-up.

## Trial population

### Screening data (21)

All GPs will complete a screening log during the recruitment period. Every patient consulting for acute gout within a quarter of the year will be recorded but remains anonymous.

### Eligibility (22)

In this pragmatic trial, eligibility will be assessed during routine care. Patients with diagnosis of gout in foot or hand will be included based on clinical presentation. This does usually not allow for consideration of new laboratory or imaging results. Thus, existing laboratory results will be used for decision making.

Inclusion criteria:

- Patients ≥ 18 years of age
- Clinical diagnosis of acute gout attack (symptoms: pain, swelling, tenderness, skin reddening or local hyperthermia)
- Acute pain in the hand or foot (podagra, chiragra)
- The onset of pain was no more than 2* calendar days ago (e.g., presentation on Monday afternoon, onset of pain on Saturday morning)
- Willingness to participate in the study and ability to give written informed consent

* prior amendment October 10, 2023: 24 h or one calendar day

Exclusion criteria:

- Known intolerance or contraindication to colchicine or prednisolone
- Known intolerance to the placebo (e.g., lactose intolerance)
- Existing or less than 2 weeks previous oral treatment with corticosteroids or colchicine
- Known chronic kidney disease (CKD stage ≥ 4) or available value of eGFR < 30 ml/min/1.73 m^2^
- Known blood formation disorder or available values of platelets < 30,000 μl or leukocytes < 4000 μl or Hb <5 mmol/l or 8 g/dl [27]
- Uncontrolled high blood pressure (systolic blood pressure permanently above 160 mmHg)
- Known liver cirrhosis or severe liver disease or available liver enzymes results (i.e., serum glutamate oxalate transaminase (GOT/ASAT) and serum glutamic pyruvic transaminase (GPT/ALAT)) with 2-fold elevation over the respective reference range
- Known current gastric or duodenal ulcer (diagnosed in the last 4 weeks)
- Current chemotherapy or chemotherapy completed less than 3 months ago
- Known HIV infection
- Solid organ transplant with immunosuppression
- Desire to have children within the next 6 months (men and women)
- Pregnancy or breastfeeding
- Participation in other studies under the German Medicines Act in the last 3 months
- Previous participation in the COPAGO trial

Exclusion after randomization:

Participants will be excluded from the study if the clinical picture deteriorates, a differential diagnosis is made (e.g., septic arthritis), unblinding is conducted due to medical emergency, or a medication for which colchicine is contraindicated has to be prescribed. Since a blood sample is taken at baseline, the eGFR value and other laboratory values are determined. If the intake of the study medication has not yet been completed and either eGFR, platelets, leukocytes, Hb, SGOT, or SGPT indicate that the exclusion criteria are met, the study participant will be contacted immediately and the intake of the study medication will be discontinued.

### Recruitment (23)

The recruitment is conducted by GPs (Fig. 2).

### Withdrawal/loss to follow-up (24a–c)

This information will be included in the study flowcharts including a tabular list for the reasons of withdrawal.

### Baseline patient characteristics (25a–b)

The baseline characteristics shown in Table 1 will be presented.

**Table 1:** Table of baseline characteristics

Characteristic	Colchicine	Prednisolone	All
*N*	na	na	na
Age, mean (SD)	na	na	na
Gender			
Male, *n* (%)	na	na	na
Female, *n* (%)	na	na	na
Non-binary, *n* (%)	na	na	na
Disease duration, years (SD)	na	na	na
< 2 years	na	na	na
2–5 years	na	na	na
6–10 years	na	na	na
> 10 years	na	na	na
Missing, *n* (%)	na	na	na
First instance of gout, *n* (%)	na	na	na
Missing, *n* (%)	na	na	na
Time since symptom onset, hours (SD)	na	na	na
Missing, *n* (%)	na	na	na
Prior pain medication (yes), *n* (%)	na	na	na
Missing, *n* (%)	na	na	na
Affected body part	na	na	na
First MTPJ, *n* (%)	na	na	na
Other foot joint, *n* (%)	na	na	na
Joint of finger and/or hand, *n* (%)	na	na	na
Missing, *n* (%)	na	na	na
No. of affected body parts			
1	na	na	na
2	na	na	na
3	na	na	na
Missing, *n* (%)	na	na	na
Pain (NRS), mean (SD)	na	na	na
Missing, *n* (%)	na	na	na
Swollen joint count, *n* (%)			
1	na	na	na
2	na	na	na
≥ 3	na	na	na
Missing, *n* (%)	na	na	na
Tender joint count, *n* (%)			
1	na	na	na
2	na	na	na
>=3	na	na	na
Missing, *n* (%)	na	na	na
Comorbidities			
Diabetes (type I/II), *n* (%)	na	na	na
Previous cardiovascular event (coronary heart disease, peripheral artery disease, stroke), *n* (%)	na	na	na
Hypertension, *n* (%)	na	na	na
Systolic blood pressure	na	na	na
Diastolic blood pressure	na	na	na
Missing, *n* (%)	na	na	na
Leukocytes (μL) mean, (SD)	na	na	na
Missing, *n* (%)	na	na	na
Platelets (nL) mean, (SD)	na	na	na
Missing, *n* (%)	na	na	na
Erythrocytes (pL) mean, (SD)	na	na	na
Missing, *n* (%)	na	na	na
Hemoglobin (mmol/L) mean, (SD)	na	na	na
Missing, *n* (%)	na	na	na
Hematocrit () mean, (SD)	na	na	na
Missing, *n* (%)	na	na	na
C-reactive protein (CRP) (mg/L) mean, (SD)	na	na	na
Missing, *n* (%)	na	na	na
Uric acid (mg/dL) mean, (SD)	na	na	na
Missing, *n* (%)	na	na	na
Creatinine (mg/dL) mean, (SD)	na	na	na
Missing, *n* (%)	na	na	na
GOT (U/l), mean, (SD)	na	na	na
Missing, *n* (%)	na	na	na
GPT (U/l), mean, (SD)	na	na	na
Missing, *n* (%)	na	na	na
eGFR (ml/min/1.73 m^2^) mean, (SD)	na	na	na
Missing, *n* (%)	na	na	na

## Analysis

The primary outcome in this randomized clinical trial is patient-reported pain after three days of treatment measured on an NRS (0–10).

### Outcome definitions (26a–c)

#### Primary efficacy endpoint

The primary outcome is measured on a NRS (0–10). Acute pain prior treatment initiation (baseline) is measured at day 0 and then repeatedly from day 1 until day 6 after treatment initiation in patient diaries. The primary outcome is evaluated at day 3 of follow-up.

#### Secondary endpoints

The secondary endpoints of this trial are the following:

- The average levels of most severe pain (last 24h) over days 1 to 6 of follow-up
- Swelling and tenderness of the joint (4-point Likert scale, day 3 after baseline)
- Physical function at day 6 compared to baseline
- Patient’s global assessment of treatment success (measured with 5-point Likert scale, day 6 after baseline)
- Most severe pain (last 24 h, measured by 11-point NRS at day 3 after baseline) depending on disease duration.
- Frequency of use of additional pain medication by treatment group.
- Frequency of use of non-pharmaceutical pain therapies in the treatment groups

### Analysis methods (27a–f)

#### Analysis of the primary outcome

For the analysis of the primary outcome, a multiple linear regression model is applied using the pain outcome at day 3 as the response. Due to adjustment for baseline values of pain, this model is sometimes referred to as an ANCOVA model [28]; it provides a similar estimate of the treatment effect as with using a *change-from-baseline* score [29].

The analysis model comprises a coefficient for the treatment allocation (prednisolone vs. colchicine, treatment ∈ (1; 0)) and several covariates measured prior drug exposure that are used for adjustment.

$$\textrm{pai}{\textrm{n}}_{d3}\sim {\beta}_0+{\beta}_1\times \textrm{treatment}+{\beta}_2\times \textrm{pai}{n}_{d0}+{\beta}_3\times \textrm{age}+{\beta}_4\times \textrm{sex}$$

with pain_*d*3_ = pain at day 3 of follow-up, pain_*d*0_ = pain at baseline, *β*_0_ = intercept, *β*_1_ = coefficient describing the adjusted treatment effect of prednisolone compared to colchicine, *β*_2_ = coefficient for the effect of pain at baseline, and *β*_*i* > 2_ = coefficients for covariates. The average difference between standard treatment (colchicine) and test treatment (prednisolone) is expressed by *β*_1_. If the upper bound of the confidence intervall for *β*_1_ is < 1, then prednicolone is considered non-inferior to colchicine. Despite the inclusion of a non-binary category for gender identity, the effect of biological sex is modeled in all analyses. There is very limited data on the prevalence of gender identities in the German adult population; one study reported no gender identification in 1.5% of adolescents [30]. In this adult population, it is expected that a maximum of 3–4 patients will state a non-binary gender. Please see the “Missing data [26]” section for the handling of the category in analyses.

Due to the possibility of missing data, the application of imputation techniques is likely. In this case, the same model will be specified; however, it will be calculated in several multiply imputed data sets. Results are then combined using SAS PROC MIANALYZE [31].

#### Analysis of secondary outcomes

For analysis of the average levels of most severe pain over days 1 to 6 of follow-up, we will apply a linear mixed effects model with random intercepts for each recruiting center to investigate the mean response between the two treatments [32]. Adjustment for covariates will be similar as for the primary outcome.

For analysis of swelling and tenderness of the joint (4-point Likert scale, day 3 after baseline), a two-sample Wilcoxon rank sum test will be applied. Similarly, for patient’s global assessment of treatment success (measured with 5-point Likert scale, day 6 after baseline).

The physical function at day 6 compared to baseline will be examined multiple linear regression model of this outcome adjusted for baseline physical limitation, age, and sex. Analysis of the most severe pain (last 24 h, measured by 11-point NRS at day 3 after baseline) depending on disease duration will be done the same model specification as for the primary outcome and substitute the adjustment for age with adjustment for disease duration.

The use of additional pain medication is examined multiple logistic regression. The outcome is defined as “use of additional medication (yes/no)” adjusted for treatment arm and pain at baseline. Similarly, for the frequency of use of non-pharmaceutical pain therapies.

#### Adjustment for covariates

In alignment with the EMA recommendations for the adjustment for baseline covariates [29], the following list of covariates will be used for adjustment in the analysis of the primary outcome: age, sex, and severity of pain at baseline. We also consider possible confounder for adjustment if imbalance between treatment arms is observed.

#### Check of assumptions

Assumptions of the linear model will be investigated. In addition, missingness pattern and associations will be examined.

#### Alternative methods

In case of missingness being completely at random, complete case analysis might be applied.

#### Sensitivity analysis

Sensitivity analyses will comprise the following aspects: (i) analysis in the per-protocol population, (ii) evaluation of the primary outcome in DECT-positive patients, (iii) if patients with multiple affected joints are less often affirmed as DECT-positive gout, and (iv) evaluation of the association between disease duration and the volume of monosodium urate crystals (under consideration of urate lowering therapy).

#### Subgroup analysis

Subgroup analyses are of exploratory nature to examine individual effect modification by subgroups. Therefore, interaction terms will be added to the analysis model one-by-one, but no further combination of interaction terms is pursued. The Holm procedure will be applied to control the type 1 error rate [33]. Conduct and reporting of subgroup analysis will adhere to recommendations [34], i.e., the overall number of conducted subgroup analyses will be reported. In addition, the exploratory nature will be highlighted as subgroups were not considered in sample size calculations.

Subgroup analyses will include, among others, DECT positive vs. DECT negative patients, elevated uric acid vs. normal or low uric acid level, elevated CRP vs. normal CRP levels, and users of pain medication prior inclusion (yes vs. now).

### Missing data (28)

The handling of missing data is aligned with EMA recommendations [35] and comprises several steps within the study design, data management, analysis, and reporting.

#### Study design

The quantity of outcomes to report in this study is kept at a minimum to avoid attrition or missing data due to patient overburden. The primary outcome and secondary outcomes are measured according to OMERACT recommendations [36] on univariate numeric rating scale (NRS: 0–10) and 4-point Likert scales which is expected to be easily applicable for patients. This rather sparse definition of outcome measures is expected to minimize the frequency of missing data.

#### Data management

Qualifying reasons for missing data will be assessed according to predefined values lists in the data dictionary. This will enable to explore further means to handle missing data. For example, paper-based records allow for deviations from expected entries; a patient may report to have *no pain* in words instead of using the NRS. In this case, queries via the study sponsor to respective GPs may rectify this data and lower the rate of missing data.

#### Analysis

In case of missing data, multiple imputation will be applied. Due to the longitudinal structure of the data, the approach of chained equations is pursued [37]. The imputation model will include all variables of the analysis model, i.e., the primary outcome pain and the covariates used for adjustment. The number of imputed data sets will be defined according to the rate of missing data in the primary outcome but will be not lower than *b* = 10 imputations [38]. The indication of a non-binary sex is considered as unknown information about the biological sex and this uncertainty will be considered during multiple imputations of missing data.

### Additional analysis (29)

Additional analyses comprise the use of rescue medication per treatment arm, the time from onset (respective gout flare) to treatment initiation on the primary outcome, and the impact of disease duration on the severity of pain at baseline.

### Harms (30)

Study patients’ adverse events will be the safety outcome of this study. All adverse events will be recorded regarding their type and severity.

Participants will be asked to report:

- Dizziness
- Nausea
- Vomiting
- Dyspepsia
- Diarrhea
- Constipation
- Abdominal pain
- Headache
- Skin rash
- Other

In addition, course of systolic blood pressure will be compared between treatments over days 1 to 6 of follow-up. We will apply a linear mixed effects model with random intercepts for each patient to investigate the mean course of systolic/diastolic blood pressure compared between the two treatments. The group allocation of patients (prednisolone vs. colchicine, treatment = (1; 0)) is the fixed effect of the model. Further adjustment is made for age, sex, and pain at baseline.

### Statistical software (31)

Statistical analysis will be done using SAS version 9.4 (SAS Institute Inc., Cary, NC, USA). For randomization and graphical illustration of study results the open source statistical software R [39] will be used.

### Reference documents (32a–d)

32a: No non-standard statistical methods will be applied 32b: Data management plan will be provided by the sponsor 32c: Trial Master File and Statistical Master File will be hosted by the sponsor 32d: Standard operating procedures are only available for DECT.

## Reporting

Reporting of this study will be according to the CONSORT statement for the reporting of clinical non-inferiority trials [40, 41].

## Data Availability

Anonymized data will be forwarded to regulators upon request.

## Abbreviations

*α*: Alpha or level of significance
AE: Adverse event
ALT: Alanine transferase
AST: Aspartate transferase
*β*: Beta or (1-*β*)*100 = power
CAD: Coronary artery disease
CRP: C-reactive protein
CTC: Common terminology criteria
CVD: Cerebrovascular disease
d: Day
*δ*: Delta of difference
DECT: Dual energy computed tomography
HbA1c: Glycated hemoglobin
eGFR: Estimated glomerular filtration rate
EMA: European medicines agency
GOT: Glutamate oxalate transaminase
GP: General practitioner
GPT: Glutamic pyruvic transaminase
H: Hypotheses
*μ*: Mu or mean
NI: Non-inferiority
NRS: Numerical rating scale
NSAIDs: Non-steroidal anti-inflammatory drugs
PVD: Peripheral vessel disease
*σ*: Sigma or standard deviation
SAE: Serious adverse event
SAP: Statistical analysis plan
ST: Standard treatment
SUSAR: Suspected unexpected serious adverse event
TT: Test treatment
VAS: Visual analogue scale

## References

[1] Richette P, Doherty M, Pascual E, Barskova V, Becce F, Castaneda-Sanabria J (2017). 2016 updated EULAR evidence-based recommendations for the management of gout. Ann Rheum Dis..

[2] Engel B, Prautzsch H, Egidi G, Stein A, Beck A, Uebel T (2013). Akute Gicht in der hausärztlichen Versorgung.

[3] van Echteld I, Wechalekar MD, Schlesinger N, Buchbinder R, Aletaha D (2014). Colchicine for acute gout. Cochrane Database Syst Rev..

[4] Finkelstein Y, Aks SE, Hutson JR, Juurlink DN, Nguyen P, Dubnov-Raz G (2010). Colchicine poisoning: the dark side of an ancient drug. Clin Toxicol..

[5] Liu X, Sun D, Ma X, Li C, Ying J, Yan Y (2017). Benefit-risk of corticosteroids in acute gout patients: an updated meta-analysis and economic evaluation. Steroids..

[6] McKenzie BJ, Wechalekar MD, Johnston RV, Schlesinger N, Buchbinder R (2021). Colchicine for acute gout. Cochrane Database Syst Rev..

[7] Lachin JM, Matts JP, Wei L (1988). Randomization in clinical trials: conclusions and recommendations. Control Clin Trials..

[8] Haff N, Choudhry NK (2018). The promise and pitfalls of pragmatic clinical trials for improving health care quality. JAMA Netw Open..

[9] Anisimov VV (2011). Effects of unstratified and centre-stratified randomization in multi-centre clinical trials. Pharm Stat..

[10] Anisimov VV, Yeung WY, Coad DS (2017). Imbalance properties of centre-stratified permuted-block and complete randomisation for several treatments in a clinical trial. Stat Med..

[11] Monaghan TF, Agudelo CW, Rahman SN, Wein AJ, Lazar JM, Everaert K (2021). Blinding in clinical trials: seeing the big picture. Medicina..

[12] Hewitt CE, Torgerson DJ (2006). Is restricted randomisation necessary?. BMJ..

[13] Snow G. Blockrand: randomization for block random clinical trials. R package version 1.5. 2020. [Available from: https://CRAN.R-project.org/package=blockrand]. last accessed: 2024-03-26.

[14] Steel GD, Robert. Relation between poisson and multinomial distributions 1953 [Available from: https://ecommons.cornell.edu/server/api/core/bitstreams/7963719a-db6f-4b05-bd4a-b918c0c17aa0/content]. last accessed: 2024-03-05.

[15] Uschner D, Schindler D, Hilgers R-D, Heussen N (2018). randomizeR: an R package for the assessment and implementation of randomization in clinical trials. J Stat Softw..

[16] International Conference on Harmonisation of Technical Requirements for Registration of Pharmaceuticals for Human Use (1999). Statistical principles for clinical trials.

[17] Chou R, Qaseem A, Snow V, Casey D, Cross JT, Shekelle P (2007). Diagnosis and treatment of low back pain: a joint clinical practice guideline from the American College of Physicians and the American Pain Society. Ann Int Med..

[18] Janssens HJ, Janssen M, Van de Lisdonk EH, van Riel PL, Van Weel C. Use of oral prednisolone or naproxen for the treatment of gout arthritis: a double-blind, randomised equivalence trial. Lancet. 2008;371(9627):1854–60. 10.1016/S0140-6736(08)60799-0. last accessed: 2024-03-05. 10.1016/S0140-6736(08)60799-0 18514729

[19] Rainer TH, Cheng CH, Janssens HJ, Man CY, Tam LS, Choi YF (2016). Oral prednisolone in the treatment of acute gout: a pragmatic, multicenter, double-blind, randomized trial. Ann Int Med..

[20] Xu L, Liu S, Guan M, Xue Y. Comparison of prednisolone, etoricoxib, and indomethacin in treatment of acute gouty arthritis: an open-label, randomized, controlled trial. Med Sci Monit: Int Med J Exp Clin Res. 2016;22(810). 10.12659/msm.895749. 10.12659/MSM.895749 PMC4791088 26965791

[21] Ahern M, Reid C, Gordon T, McCredle M, Brooks P, Jones M (1987). Does colchicine work? The results of the first controlled study in acute gout. Aust N Z J Med..

[22] Terkeltaub RA, Furst DE, Bennett K, Kook KA, Crockett R, Davis MW (2010). High versus low dosing of oral colchicine for early acute gout flare: twenty-four–hour outcome of the first multicenter, randomized, double-blind, placebo-controlled, parallel-group, dose-comparison colchicine study. Arthritis Rheum..

[23] Roddy E, Clarkson K, Blagojevic-Bucknall M, Mehta R, Oppong R, Avery A (2020). Open-label randomised pragmatic trial (CONTACT) comparing naproxen and low-dose colchicine for the treatment of gout flares in primary care. Ann Rheum Dis..

[24] Vollset SE (1993). Confidence intervals for a binomial proportion. Stat Med..

[25] Food and Drug Administration FDA (2016). Non-inferiority clinical trials to establish effectiveness: guidance for industry.

[26] Bai AD, Komorowski AS, Lo CKL, Tandon P, Li XX, Mokashi V (2021). Intention-to-treat analysis may be more conservative than per protocol analysis in antibiotic non-inferiority trials: a systematic review. BMC Med Res Methodol..

[27] World Health Organization (2011). Haemoglobin concentrations for the diagnosis of anaemia and assessment of severity.

[28] Vickers AJ, Altman DG (2001). Analysing controlled trials with baseline and follow up measurements. BMJ..

[29] Committee for Medicinal Products for Human Use (2013). Guideline on adjustment for baseline covariates in clinical trials.

[30] Becker I, Ravens-Sieberer U, Ottová-Jordan V, Schulte-Markwort M (2017). Prevalence of adolescent gender experiences and gender expression in Germany. J Adolesc Health..

[31] Yuan Y. Multiple imputation using SAS software. J Stat Softw. 2011;45(6):1-25–1. 10.18637/jss.v045.i06. last accessed: 2024-03-05.

[32] Verbeke G, Molenberghs G (2009). Linear mixed models for longitudinal data.

[33] Holm S (1979). A simple sequentially rejective multiple test procedure. Scand J Stat..

[34] Wang R, Lagakos SW, Ware JH, Hunter DJ, Drazen JM (2007). Statistics in medicine--reporting of subgroup analyses in clinical trials. N Engl J Med..

[35] Committee for Medicinal Products for Human Use (2010). Guideline on missing data in confirmatory clinical trials.

[36] Singh JA, Taylor WJ, Dalbeth N, Simon LS, Sundy J, Grainger R (2014). OMERACT endorsement of measures of outcome for studies of acute gout. J Rheumatol..

[37] Liu Y, De A. Multiple imputation by fully conditional specification for dealing with missing data in a large epidemiologic study. Int J Stat Med Res. 2015;4(3):287–95. 10.6000/1929-6029.2015.04.03.7. last accessed: 2024-03-05. 10.6000/1929-6029.2015.04.03.7 PMC4945131 27429686

[38] Sterne JAC, White IR, Carlin JB, Spratt M, Royston P, Kenward MG (2009). Multiple imputation for missing data in epidemiological and clinical research: potential and pitfalls. BMJ..

[39] R Development Core Team (2023). R: a language and environment for statistical computing.

[40] Schulz KF, Altman DG, Moher D, the CG (2010). CONSORT 2010 Statement: updated guidelines for reporting parallel group randomised trials. Trials..

[41] Piaggio G, Elbourne DR, Pocock SJ, SJW E, Altman DG, Consort Group ft (2012). Reporting of noninferiority and equivalence randomized trials: extension of the CONSORT 2010 statement. Jama..

